# Is the new ASNM intraoperative neuromonitoring supervision “guideline” a trustworthy guideline? A commentary

**DOI:** 10.1007/s10877-018-00242-3

**Published:** 2019-01-05

**Authors:** Stanley A. Skinner, Elif Ilgaz Aydinlar, Lawrence F. Borges, Bob S. Carter, Bradford L. Currier, Vedran Deletis, Charles Dong, John Paul Dormans, Gea Drost, Isabel Fernandez-Conejero, E. Matthew Hoffman, Robert N. Holdefer, Paulo Andre Teixeira Kimaid, Antoun Koht, Karl F. Kothbauer, David B. MacDonald, John J. McAuliffe, David E. Morledge, Susan H. Morris, Jonathan Norton, Klaus Novak, Kyung Seok Park, Joseph H. Perra, Julian Prell, David M. Rippe, Francesco Sala, Daniel M. Schwartz, Martín J. Segura, Kathleen Seidel, Christoph Seubert, Mirela V. Simon, Francisco Soto, Jeffrey A. Strommen, Andrea Szelenyi, Armando Tello, Sedat Ulkatan, Javier Urriza, Marshall Wilkinson

**Affiliations:** 1 Intraoperative Neurophysiology Department, Abbott Northwestern Hospital, Minneapolis, MN USA; 2 Acibadem University School of Medicine, Department of Neurology, Istanbul, Turkey; 3 Massachusetts General Hospital, Harvard Medical School, Boston, MA USA; 4 Department of Neurosurgery, Harvard Medical School, Massachusetts General Hospital, Boston, MA USA; 5 Department of Orthopedics, Mayo Clinic, Rochester, MN USA; 6 Albert Einstein College of Medicine, New York, NY USA; 7 Division of Neurosurgery, Department of Surgery, University of British Columbia, Vancouver, BC Canada; 8 Pediatric Orthopedic Surgery, Riley Hospital for Children, Indiana University School of Medicine, Indianapolis, IN USA; 9 Department of Neurosurgery/Neurology, University Medical Center Groningen, Groningen, The Netherlands; 10 Department of Intraoperative Neurophysiology, Hospital Universitari de Bellvitge, L’Hospitalet de Llobregat, Barcelona, Spain; 11 Department of Neurology, Mayo Clinic, Rochester, MN USA; 12 Department of Rehabilitation Medicine, University of Washington, Harborview Medical Center, Seattle, WA USA; 13 Department of Neurosurgery, Federal University of São Paulo, São Paulo, Brazil; 14 Feinberg School of Medicine, Northwestern University, Chicago, IL USA; 15 Division of Neurosurgery, Luzerner Kantonsspital, University of Basel, 6000 Lucerne, Switzerland; 16 Department of Neurosciences, Section of Neurophysiology, King Faisal Specialist Hospital & Research Center, MBC 76, PO Box 3354, Riyadh, 11211 Saudi Arabia; 17 Department of Anesthesia, Cincinnati Children’s Hospital, The University of Cincinnati, Cincinnati, OH USA; 18 Neurostatus, Eagle, ID USA; 19 IWK Children’s Health Program & Division of Neurosurgery, Dalhousie University, Halifax, NS Canada; 20 Department of Surgery, University of Saskatchewan, Saskatoon, SK Canada; 21 Department of Neurosurgery, Medical University of Vienna, Vienna, Austria; 22 Department of Neurology, Seoul National University Bundang Hospital, Seoul National University College of Medicine, Seongnam, Republic of Korea; 23 Twin Cities Spine Center, Minneapolis, MN USA; 24 Department of Neurosurgery, Halle (Saale), Germany; 25 Neurophysiology Department, Abbott Northwestern Hospital, Minneapolis, MN USA; 26 Section of Neurosurgery, Department of Neurosciences, Biomedicine and Movement Sciences, University Hospital, Verona, Italy; 27 Surgical Monitoring Associates, Springfield, PA USA; 28 Clinical Neurophysiology Unit, Hospital Garrahan, University of Buenos Aires Medical School, Buenos Aires, Argentina; 29 Intraoperative Neurophysiology and Neuro-oncological Surgery, Department of Neurosurgery, Inselspital Bern University Hospital, Bern, Switzerland; 30 Division of Neuroanesthesia, College of Medicine, University of Florida, Gainesville, FL USA; 31 Intraoperative Neurophysiology Unit, Department of Neurology, Harvard Medical School, Massachusetts General Hospital, Boston, MA USA; 32 Neurology Department, Clínica Las Condes, Santiago, Chile; 33 Department of Physical Medicine and Rehabilitation, Mayo Clinic, Rochester, MN USA; 34 Neurophysiology Department, Abbott Northwestern Hospital, Minneapolis, MN USA; 35 Clinical and Intraoperative Neurophysiology, Department of Neurosurgery, Hospital of University of Munich, Ludwig Maximilians Universitaet Muenchen (LMU), Munich, Germany; 36 Clinical Neurophysiology Department, Hospital Español, Mexico City, Mexico; 37 Intraoperative Monitoring Service, Mount Sinai West Hospital, New York, NY USA; 38 Intraoperative Neurophysiology Unit, Clinical Neurophysiology Department, Complejo Hospitalario de Navarra – B, Pamplona-Iruña, Navarra, Spain; 39 Section of Neurosurgery, University of Manitoba, Winnipeg, MB Canada

## Introduction: provider-centered versus patient-centered IONM supervision

The new ASNM intraoperative neuromonitoring (IONM) supervision guideline [1] attempts to justify current remote IONM practices.^1^ Contrary to ordinary clinical practice guideline development, it is provider-centered, not patient-centered.

The new guideline could have embraced recent scholarship that indicates the need to provide robust teamwork and medical error avoidance. It could have moved in the direction of improved patient safety and outcomes. Instead, key words are repeated (“communicate,” “collaborate,” “team”) many times, but with no meaningful strategy to achieve their patient safety potential. In fact, within this new guideline, an IONM remote provider’s communication with in-room physician peers and co-practitioners is defined as: “… at minimum, direct voice access (via ‘land-line’ or cellular network) for perioperative communication with the surgical team”.

## The evidence for optimized intraoperative team communication and decision-making

Two-thirds of medical errors derive from poor team performance during patient care [2, 3]. Such errors are lessened with rigorous adherence to *situational awareness* and *critical language* [4]. “Talk among physicians is essential in the negotiation of professional relationships, the distribution of responsibility, the inducement of cooperation, and the assessment of competence [5].” And, “The current weaknesses in communication in the OR may derive from a lack of standardization and team integration… decisions are often made without all relevant team members present, and much communication is consequently reactive and tension provoking [6].” Furthermore, scholars increasingly recommend using all means to reduce errors and emphasize *team familiarity* as a major element contributing to patient safety [7, 8].

Improved outcomes with IONM depend on timely and appropriate surgeon responses to IONM alerts [9–11]. The assertion that an unseen, distant, and often unknown interpreter can effectively discuss the implications of an IONM alarm during crisis management should be questioned. The added expertise and counsel provided by the supervising IONM provider allows the surgeon to weigh the risks and benefits of the available options. It is highly unlikely that an off-site provider, available only by phone, could provide the same value in these high-risk and stressful situations.

Based on a systematic review of operating room teamwork, communication, and safety, “[Operating room] culture improvement appears to be associated with… positive effects, including better patient outcomes” [12]; support for this conclusion appears in a separate review [13]. Furthermore, the World Health Organization’s Guidelines for Safe Surgery stipulate all the above prerequisites, again based on systematic review [14].

Clearly, there is substantial evidence that the most direct flow of information between all intraoperative team members is an important factor to ensure quality patient care. Thus, it is reasonable to assume that the limited communication inherent to remote IONM may adversely affect quality and patient outcomes. Since the new guideline provides no evidence that this is not the case, it should not recommend a voice access minimum standard.

## Remote IONM is not the virtual patient care practiced in teleStroke or teleICU

Because remote IONM is telecommunication based, a pertinent review of virtual medical care would have been helpful. However, the new guideline reflects unawareness of the benefits of enriched telecommunication versus the harms of poor telecommunication. For example, controlled trials comparing phone-based to audiovisually enriched teleStroke care have shown that patient outcomes are poorer using phone based communication [15–17]. Also, a major teleICU controlled study revealed that patient outcomes are similar when *audiovisually enriched virtual care* is compared to personal care [18].

Unfortunately, current remote IONM practices exemplify poor telecommunication. These essential questions were raised in 2010 [19]: “How can an individual outside the OR interpret fluctuating neuromonitoring data and develop explanations of cause? How it is possible to verify an appropriate level of vigilance on the part of the remotely connected professional, particularly if multiple cases are being monitored simultaneously?” To date, no answers have been forthcoming.

But the new guideline further asserts that with a phone connection and internet waveform display, the supervising neurophysiologist effectively: “Communicates and collaborates with other members of the patient care team”; “Evaluates IONM data in the context of the procedure”; “Evaluates and interprets data obtained from topographical/neuro-navigation studies”; and “determines if changes are related to iatrogenic injury, anesthetic effects, physiological variables, patient positioning, technical factors, or a combination of these”.

One may extend the concerns unanswered since 2010. How is it possible to achieve these duties without personal or virtual situational awareness? How is it possible to make credible recommendations during a situationally unaware phone call between practitioners who are barely or not at all known to each other?

The new guideline does stipulate: “It is further recognized that cases of greater complexity may require personal attendance in the operating room.” What are those cases? For any IONM case of any complexity, which of the duties listed above can be routinely addressed by a phone connection?

## Remote IONM and patient care

The new guideline states that IONM supervision “constitutes a patient care activity”, but its actual language does not support this role: “Guideline authors recommend direct or indirect interaction with the patient to the extent possible. This can be accomplished through multiple pathways, including a collaboration with the IONM-T [technologist]”. The Centers for Medicare & Medicaid Services (CMS) stipulate that telemedicine services are “provided to the patient in ‘real time’ by the telemedicine practitioner, similar to the actions of an on-site practitioner when called in by a patient’s attending physician to see the patient” [15, 20]; there is no intermediary nurse or technologist.

Remote providers do not engage in either direct or virtual patient care by the definitions of CMS or by best practices within tele-ICU or teleStroke care. Therefore, it would have been more forthright to simply state that the remote provider interprets waveforms and e-chats with the technician/technologist or telephones other staff as needed. At best, that is what really happens during most remote IONM. This non-patient care role has also been defined by CMS to cover tele-radiology, for example [20]. Potentially effective tools to achieve virtual IONM patient care are readily available, but are not stipulated in the new guideline.

## Stakeholder engagement

There are multiple stakeholders in the dialogue occurring during IONM. The new guideline fails to account for the perspective of surgeons, anesthesiologists, or patients about the limitations of the remote monitoring approach. The new guideline does not include support for the proposed recommendations from the surgeon or the anesthesiology community. This feedback should have been sought and broadly developed before proceeding. A consensus approach would develop true guidelines as to the specific nature of the remote IONM activity addressing such issues as: telephone/chat only versus audiovisually enhanced IONM, distractibility issues including number of cases permitted to be simultaneously monitored, and the physical location of the remote monitoring physician. Just as surgeons and anesthesiologists have immediate physical availability requirements in the OR environment, supervising IONM providers, as key members of the operative team, should be held to similarly high standards.

## Guideline or position statement?

The original 2014 ASNM supervision guideline [21] was drafted before the Institute of Medicine’s 2011 “Clinical Practice Guidelines We Can Trust” [22] became a widely accepted standard. In retrospect, the 2014 document was actually a position statement. Certainly, since 2014, Institute of Medicine standards should have been followed. These stipulate that, “To be trustworthy, guidelines should be based on a systematic review of the existing evidence”, and that “Clinical practice guidelines fundamentally rest on appraisal of the quality of relevant evidence, comparison of the benefits and harms of particular clinical recommendations, and value judgments regarding the importance of specific benefits and harms.” While the new guideline states that ASNM “periodically publishes Clinical Practice Guidelines consistent with the Institute of Medicine,” it fails to meet any of these standards. It may be a position statement, but it is not a clinical practice guideline.

The new guideline offers an unsound justification for an update and “over-write” of the original 2014 supervision guideline: “… that [2014] document has undergone review and revision to accommodate broad inter- and intra-societal feedback.” In fact, updating guidelines, under the Institute of Medicine’s report, is a formal task: “Changes in evidence, the values placed on evidence, the resources available for health care, and improvements in current performance are all possible reasons for updating clinical guidelines [22, 23].” Neither majority societal opinion nor the fraction of clinicians currently meeting a systematized evidential criterion creates a legitimate basis for a guideline update or replacement.

## Conclusion

The new IONM supervision “guideline” asserts that routine phone-based communication with the surgeon and other members of the operating room team meets a minimum expectation, but offers no supportive empirical evidence. Crucial surgeon and anesthesiology stakeholders were not consulted. It does not conform to Institute of Medicine standards; it does not provide systematized evidence, benefit/harms analysis, or disclosure(s) of potential conflicts of interest with the remote monitoring industry. Therefore, by Institute of Medicine criteria, the article is not a guideline.

Unfortunately, the literature demonstrating that optimized operating room collaboration avoids surgical errors has been ignored. The telemedicine literature recommending enhanced audiovisual connectivity in high risk environments like the ICU and during teleStroke care has been excluded. The new guideline may be interpreted as a license for maintenance of the status quo, thereby inhibiting the adoption of new technologies that have the potential to elevate the quality of remote monitoring.

Although “communication” and “collaboration” and “patient care” are rhetorically supported, no effective mechanisms are described to realize these patient-centered goals. Effective communication within multidisciplinary teams does not start and end with a case. It is increasingly acquired over years of collaborative work.

The new “guideline” appears to be chiefly aimed at protecting the business model of the remote monitoring industry. Surgeons, hospitals, payers, and the broader IONM community may wish to assess the implications of its many flawed premises.

## Footnotes

1 By remote IONM, we mean the dominant US model of IONM in which the supervising neurophysiologist is often unfamiliar with, distant from, and unavailable to in-room colleagues (either personally or virtually) and is situationally unaware. Local hub to satellite “remote” IONM of less complex cases and among familiar team members can be fraught with diminished situational awareness, but is not the focus of this commentary.
